# Autologous adipose-derived stem cells for the treatment of complex cryptoglandular perianal fistula: a prospective case-control study

**DOI:** 10.1186/s13287-020-01995-y

**Published:** 2020-11-10

**Authors:** Yang Zhang, Min Ni, Chungen Zhou, Yehuang Wang, Yaxian Wang, Yang Shi, Jing Jin, Rui Zhang, Bin Jiang

**Affiliations:** 1 National Colorectal Disease Center of Nanjing Hospital of Chinese Medicine Affiliated to Nanjing University of Chinese Medicine, Nanjing, 210022 Jiangsu Province China; 2 Graduate School of Nanjing University of Chinese Medicine, Nanjing, 210029 Jiangsu Province China; 3 Research Institute of Jiangsu Decon Bio-science Technologies Company Ltd., Nanjing, 210000 Jiangsu Province China

**Keywords:** Autologous adipose-derived stem cells, Endorectal advancement flap, Complex cryptoglandular perianal fistula, Efficacy, Safety

## Abstract

**Background:**

Complex cryptoglandular perianal fistula (CPAF) is a kind of anal fistula that may cause anal incontinence after surgery. Minimally invasive surgery of anal fistula is constantly emerging. Over the past 20 years, there are several sphincter-sparing surgeries, one of which is autologous adipose-derived stem cell (ADSC) transplantation. However, to date, there is no study regarding the treatment of complex CPAF with ADSC in China. This is the first study in China on the treatment of complex CPAF with ADSC to evaluate its safety and efficacy.

**Methods:**

Totally, 24 patients with complex CPAF were enrolled in this prospective case-control study from January 2018 to December 2019 in the National Colorectal Disease Center of Nanjing Hospital of Chinese Medicine Affiliated to Nanjing University of Chinese Medicine. Patients were divided into ADSC group and endorectal advancement flap (ERAF) group according to their desire. The healing of fistulas (healing of all treated fistulas at baseline, confirmed by doctor’s clinical assessment and magnetic resonance imaging or transrectal ultrasonography) was evaluated at week 12 after treatment. In addition to their safety evaluation based on adverse events monitored at each follow-up, the patients were also asked to complete some scoring scales at each follow-up including pain score with visual analog score (VAS) and anal incontinence score with Wexner score.

**Results:**

The closure rates within ADSC group and ERAF group at week 12 were 54.55% (6/11) and 53.85% (7/13), respectively, without significant difference between them. VAS score in ADSC group was significantly lower than that in ERAF group at the 5th day postoperatively [1(0,2) VS 2(2,4), *p* = 0.011], but no differences were observed at the other time. Wexner score of all patients was not increased with no significant differences between the two groups. Adverse events were observed fewer in ADSC group (27.27%) than that in ERAF group (53.85%), but there was no significant difference between them.

**Conclusion:**

This study indicated safety and efficiency of ADSC for the treatment of complex CPAF in the short term, which is not inferior to that of ERAF. ADSC may provide a promised and potential treatment for complex CPAF conforming to the future of the treatment, which is reconstruction and regeneration.

**Trail registration:**

ChiCTR, ChiCTR1800014599. Registered 23 January 2018—retrospectively registered, http://www.chictr.org.cn/showproj.aspx?proj=24548

## Introduction

Perianal fistula is one of the common colorectal diseases with an incidence rate between 1.1 and 2.2 per 10,000 persons per year, which is caused mainly by infection of anal glands [1, 2]. Although most patients can be cured by surgical operation, the treatment of complex anal fistula remains a big challenge because of high lesion location, pipe bend, and dead space, with a high rate of recurrence and frequent side effects, especially as fecal incontinence [3–5]. Minimally invasive surgery of anal fistula is an emergence, and there are several sphincter-sparing surgeries in the past 20 years, but the most suitable surgical treatment has not been determined [6, 7].

**Fig. 1:**
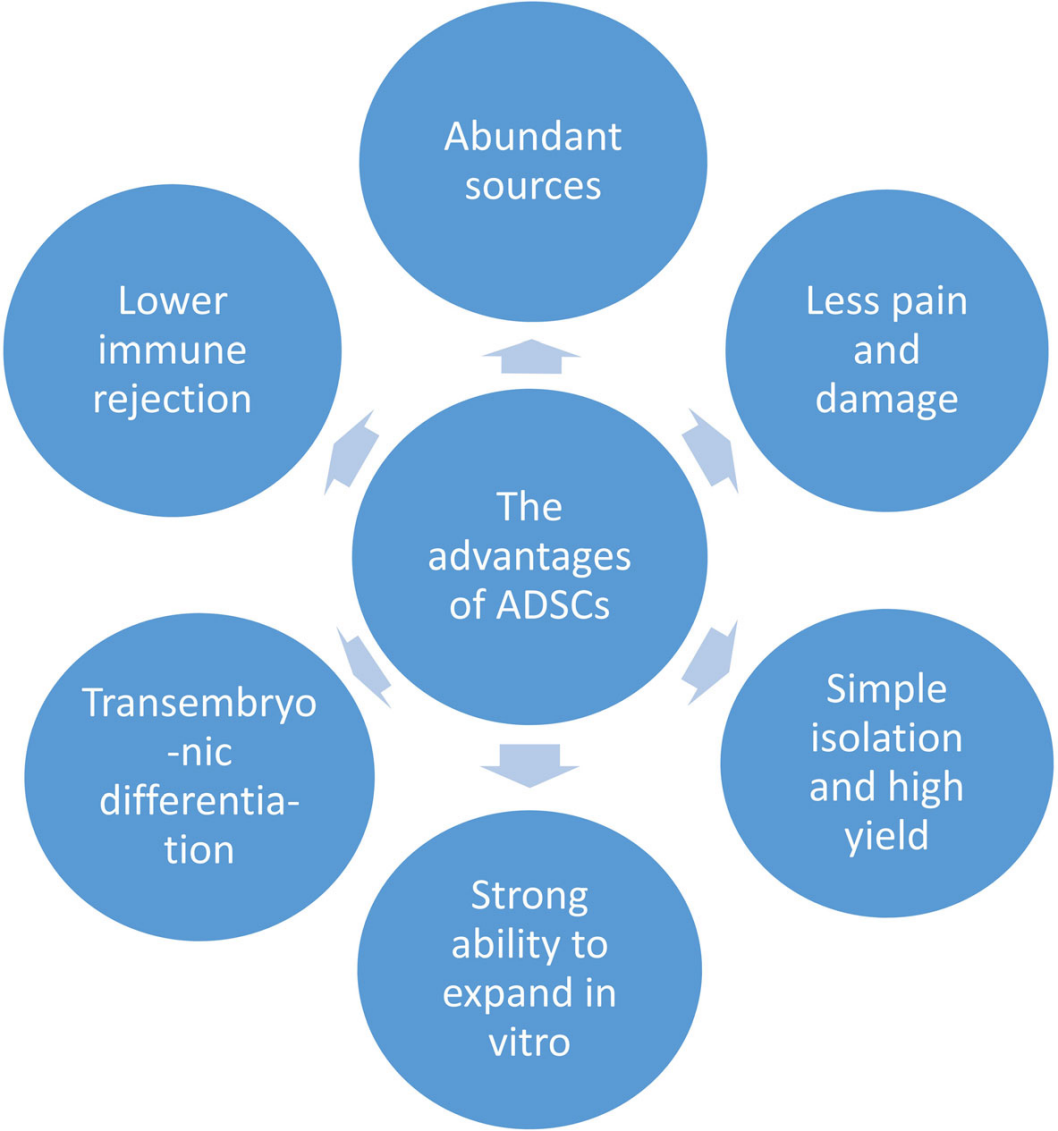
The advantages of ADSCs

Adipose-derived stem cell (ADSC) is a population of pluripotent cells derived from adipose tissue [8]. It has been widely used in clinical practice [9, 10] including diabetic foot, inflammatory bowel disease, and osteoarthritis, because of its many advantages compared with other sources of stem cells (Fig. 1) [11–13]. Recently, ADSC, a new biotechnology, has also been reported as a safe and effective treatment of complex cryptoglandular perianal fistula (CPAF) in several previously published literatures (Table 1) [14–19]. We previously reported the experience of ADSC in the treatment of Crohn’s fistula-in-ano and concluded that it has the following advantages: protecting anal function of patients, relieving pain, allowing a quick recovery, being well-tolerated, and improving the quality of life during postoperative period [20]. Furthermore, we conducted a clinical study on ADSC in the treatment of complex CPAF. The aim of this study was to evaluate the efficacy and safety of autologous ADSCs for treatment of complex CPAF compared with the operation of ERAF.

**Table 1:** The situation of ADSC in the treatment of CPAF

Author	Year	Country	Therapy	Number of cases	Follow-up time	Healing rate (%)	Incontinence rate (%)
Garcia-Olmo D et al. [14]	2009	Spain	ADSC+FG	17	8 weeks	70.59	0
Herreros MD et al. [15]	2012	Spain	A: ADSC B: ADSC+FG	A: 64 B: 60	1 year	A: 57.1 B: 52.4	0
Borowski DW et al. [16]	2012	England	ADSC+ERAF	3	2 to 3 years	100	0
Choi S et al. [17]	2017	Korea	ADSC	13	8 weeks	69.2	0
Topal U et al. [18]	2019	Turkey	ADSC	10	9 months	70	0
Garcia-Arranz M et al. [19]	2020	Spain	CPAF	23	2 years	50.0	0

*FG* fibrin glue

## Materials and methods

### Study population

**Fig. 2:**
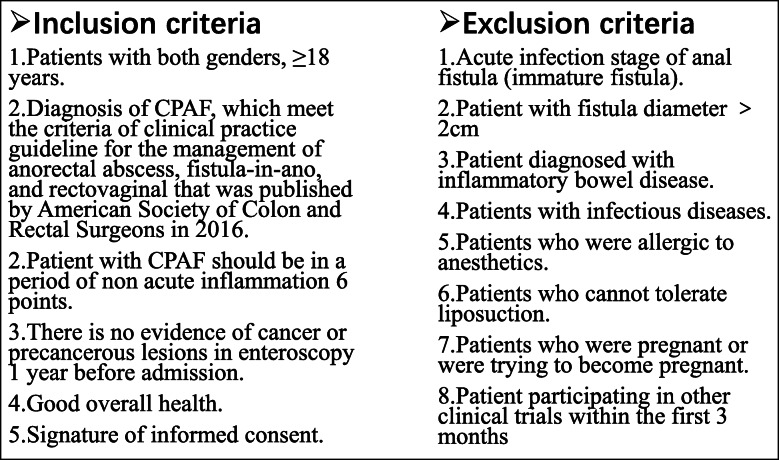
Inclusion and exclusion criteria

Eligible participants were adult patients with complex CPAF hospitalized in Nanjing Hospital of Chinese Medicine from January 2018 to December 2019. The inclusion and exclusion criteria were presented in Fig. 2.

### Study protocol

We designed and conducted according to the principles of the Declaration of Helsinki. This study was reviewed and approved by the Ethics Review Committee of Nanjing Hospital of Chinese Medicine (Ethics Review No. KY2018011) and was registered in the China Clinical Trials Registry (No. ChiCTR1800014599). Written informed consent was obtained from each patient before enrollment.

**Fig. 3:**
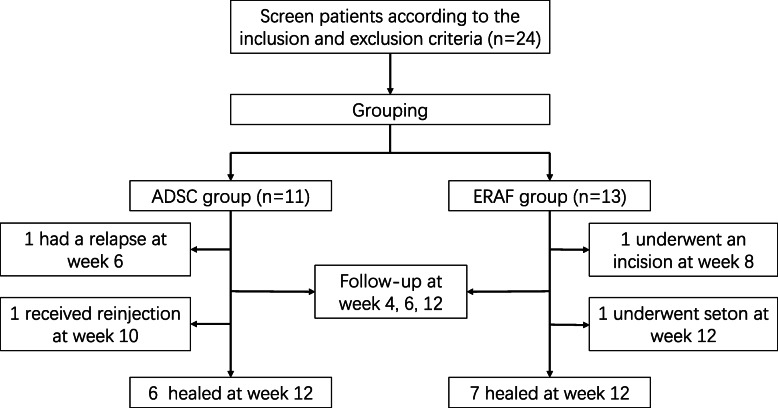
Flow chart of the study

This study was a prospective case-control study to evaluate the safety and efficacy of ADSC in the treatment of complex CPAF. The patients were divided into ADSC group and ERAF group according to their desire. All the patients in the ADSC group received ADSC treatment, while patients in the ERAF group received ERAF treatment. Each patient was followed up for a minimum of 12 weeks after the last treatment to evaluate the safety and efficacy. Figure 3 shows the flow chart of the study.

### Treatment of ADSC group

**Fig. 4:**
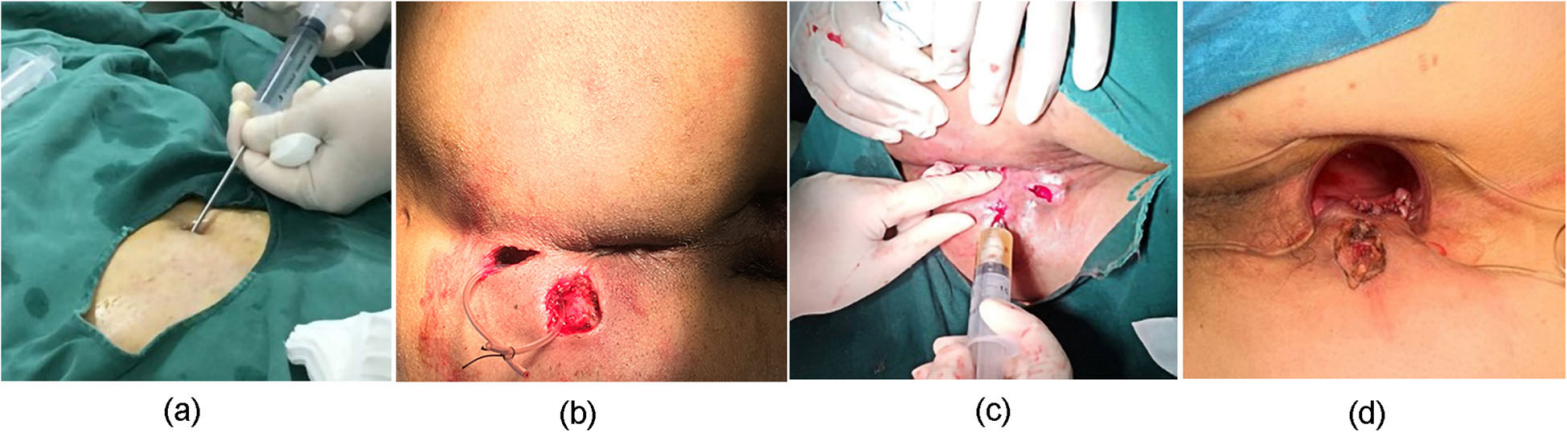
Treatment of patients. **a** Liposuction. **b** Fistula preparation. **c** Injection of ADSCs. **d** Suture of ERAF

**Fig. 5:**
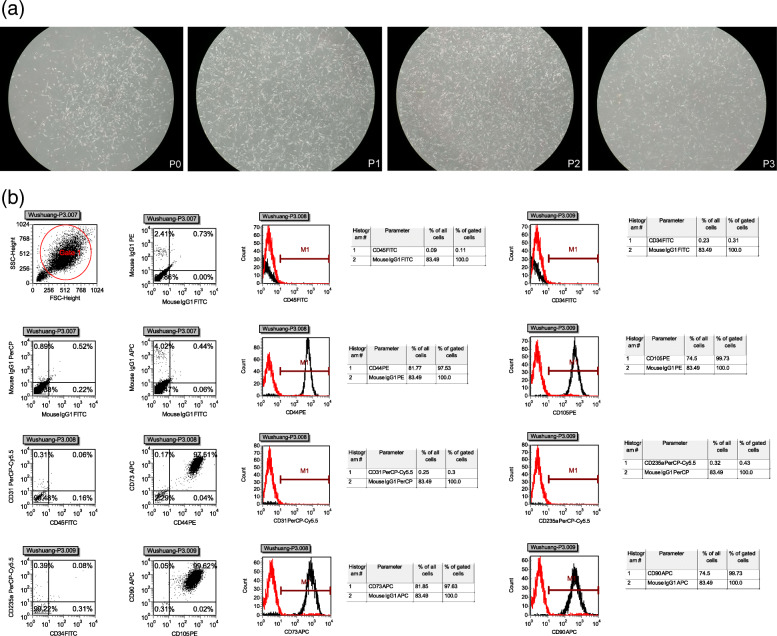
Preparation of ADSCs. **a** Cell morphology of each generation. **b** Cell phenotype of ADSCs

The procedure of liposuction (Fig. 4a), fistula preparation (Fig. 4b), preparation of ADSCs (Fig. 5), and injection of ADSC (Fig. 4c) is the same as that of ADSC treatment of Crohn’s fistula-in-ano which we have reported [20].

Firstly, a plastic surgeon used the “syringe liposuction” introduced by Pierre Fournie [21] to suck fat from the patient’s lower abdomen or inner and outer thighs under swelling anesthesia. In the immediate aftermath of the liposuction, patients received fistula preparation more than 2 weeks before ADSC injection, which included fistula exploration, curettage, and drainage with seton. Then, the fat was separated, cultured, proliferated, and identified for 2 weeks at Jiangsu Decon Bio-science Technologies Company Ltd. Next, all the epithelial tissues of the fistulas were removed, and internal openings were closed with 2–0 Vicryl. Subsequently, ADSCs with the concentration of 5 × 10^6^ cells/ml were injected into all quadrants around the internal opening and tract of fistulas repeatedly (> 4 times) and uniformly according to the diameter and length of fistulas measured by MRI and clinical evaluation. One milliliter of ADSCs/cm was injected when the diameter was less than 1 cm, and 2 ml ADSCs/cm was injected with the diameter ranging from 1 and 2 cm. Finally, ADSCs with the concentration of 1 × 10^6^ cells/ml were infused into the fistula and the external opening was closed. In addition, if the fistula had not healed at 8 weeks, the patient would receive a second treatment according to the same protocol, who agreed to receive injection again with the concentration of 1 × 10^7^ cells/ml.

### Treatment of ERAF group

This technique was performed under intraspinal anesthesia with the patient in the lateral position. The full-thickness flap, which consists of mucosa, submucosa, and some circular muscle fibers, was moved 4–5 cm from the dentate line to the cephalic side. The bottom of the flap was at least twice as wide as its top to ensure an adequate blood supply to the distal tissue. After the resection of the distal part of the flap with the internal opening and the curettage of the fistula, the internal portion of the fistula was sutured with 2–0 Vicryl in figure-of-eight suture. The flap was sutured without tension to cover the internal opening with 2–0 Vicryl. (Fig. 4d).

### Assessments

The same variable definitions and evaluation procedures were applied to evaluate the safety and efficacy for all participants in both intervention and control groups.

### Efficacy

If the fistula had not healed at 8 weeks, the patient received a second dose of fibrin glue or fibrin glue plus ASCs (twice the dose of the first treatment, 4 [times] 10 million) and was reassessed according to the same protocol.

**Fig. 6:**
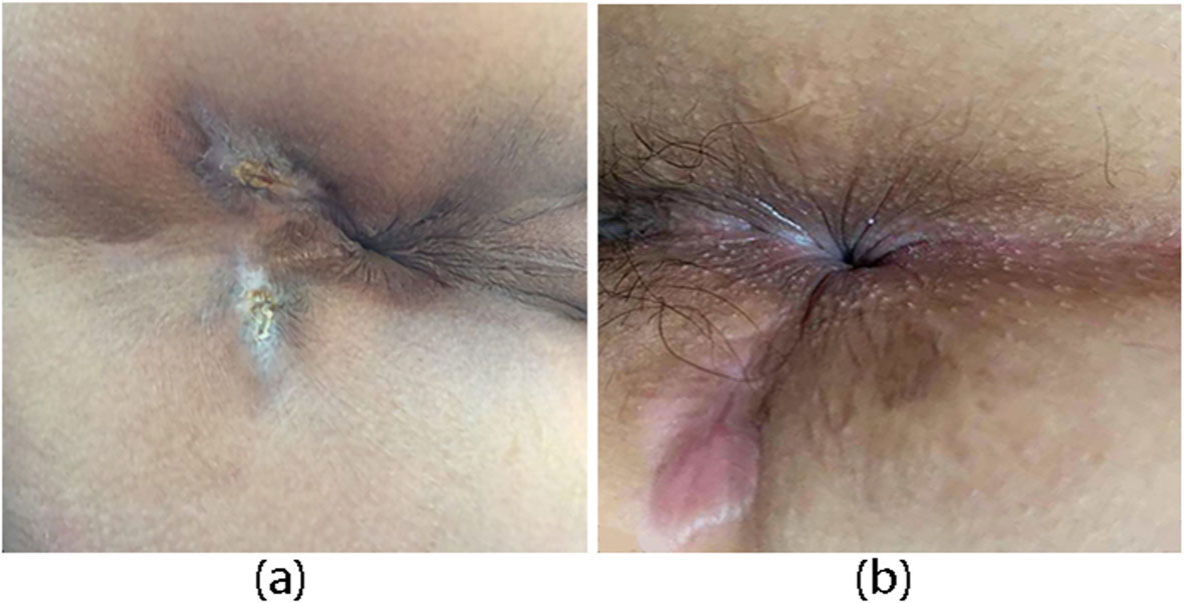
The closure of the fistula at week 12. **a** The closure of the fistula with ADSC. **b** The closure of the fistula with ERAF

The primary endpoint for efficacy was defined as the proportion of patients whose fistula was closed at week 12 postoperatively. The closure of the fistula was evaluated through clinical evaluation and magnetic resonance imaging (MRI) or endorectal ultrasonography (ERUS) at 12 weeks postoperatively. Closure was defined as the complete epithelialization of external openings (i.e., no pus outflow from the external openings under any circumstances, Fig. 6) and no evidence of fistulas in MRI or ERUS.

The secondary endpoints for efficacy included pain score with visual analog score (VAS) and anal incontinence score with Wexner score, which were completed by patients at each follow-up.

### Safety

Safety was assessed by determining the incidence of adverse events (AEs) and serious AEs (SAEs). During each follow-up, the AEs of patients were monitored. AEs included fever (axillary temperature > 38 °C), perianal pain (VAS score > 5 points), anal distention, uroschesis, and secretion.

### Statistical analyses

The continuous measurements, obeying normal distribution, were presented with means and SD; otherwise, they were described with median (quartile) [M (p25, p75)]. For categorical variables, they were described as percentages and 95% confidence interval (CI). *T* test or non-parametric test was used to make comparisons of continuous variables between sub-groups, while chi-square test or Fisher exact test was applied to make comparisons for categorical measures in this study. *P* < 0.05 was considered statistically significant in all statistical tests. All statistical analyses were performed with SPSS 22.0 software.

## Results

### Patients characteristics

Among the 24 patients, there were 4 women and 20 men, with a mean (standard deviation, SD) age of 34.25 ± 7.97 years. There was no significant difference in selected characteristics between the two groups at baseline (Table 2). All the patients in both groups completed the 12-week follow-ups. The average volume of fat extracted from the patients in ADSC group was 52.73 ± 28.23 mL and number of ADSCs was (89.70 ± 57.42) × 10^6^ cells.

**Table 2:** Characteristics of patients at baseline

Contents	ADSC group(***n*** = 11)	ERAF group(***n*** = 13)
**Sex**		
Male	10 (90.91%)	10 (76.92%)
Female	1 (9.09%)	3 (23.08%)
**Age (year)**	35.73 ± 7.54	33.00 ± 8.42
**BMI (kg/m**^**2**^**)**	25.30 ± 4.98	23.91 ± 2.98
**Volume of fat (mL)**	52.73 ± 28.23	–
**Number of cells (◊10**^**6**^**cells)**	89.70 ± 57.42	–
**VAS**^**§**^	0.8 ± 1.0	1.1 ± 1.0
**Wexner**^**¶**^	1.2 ± 1.3	1.5 ± 0.7
**Classification of CPAF (Parks)**		
Transsphincteric	5 (45.45%)	7 (53.85%)
Suprasphincteric	6 (54.55%)	6 (46.15%)

Data are mean (SD) or number (%). Percentages might not always add up to exactly 100% as a result of rounding. *BMI* body mass index. ^§^Score ranges from 0 to 10; higher scores suggest more severe degree of pain. ^¶^Score ranges from 0 to 20; higher scores suggest more severe degree of anal incontinence

### Efficacy

#### Primary endpoint

**Fig. 7:**
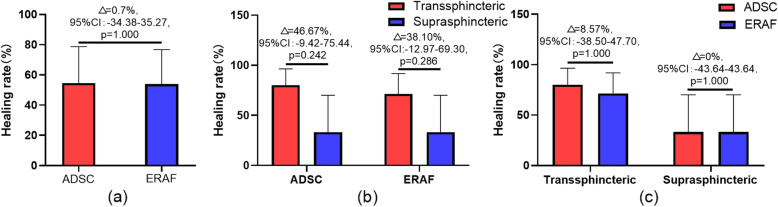
Primary endpoint. **a** Closure rate at week 12. **b** Closure rate of different types of CPAF in each group. **c** Closure rate of different types of CPAF

At week 12, there were no significant differences between ADSC group and ERAF group (6/11 [54.55%] vs 7/13 [53.85%], difference [95% CI] 0.7 percentage points [− 34.38 to 35.27], *p* = 1.000; Fig. 7a and Table 3). There were also no significant differences between the different types of CPAF in each group (4/5 [80.00%] vs 2/6 [33.33%], 5/7 [71.43%] vs 2/6 [33.33%], respectively; difference [95% CI] 46.67 percentage points [− 9.42 to 75.44], *p* = 0.242; and 38.10 percentage points [− 12.97 to 69.30], *p* = 0.286; Fig. 7b). In addition, the difference in the closure rate between the two stratification groups was also not significant (4/5 [80.00%] vs 5/7 [71.43%], 2/6 [33.33%] vs 2/6 [33.33%], respectively; difference [95% CI] 8.57 percentage points [− 35.80 to 47.70], *p* = 1.000 and 0.00 percentage points [− 43.64 to 43.64], *p* = 1.000; Fig. 7c and Table 3).

**Table 3:** Primary endpoint

Patients	ADSC			ERAF			Analysis		
	Total	No. closed	%	Total	No. closed	%	Difference	95% CI	*P* value
**All cases**	11	6	54.55	13	7	53.85	0.7	− 34.38–35.27	1.000
**Subgroup analyze**									
Transsphincteric	5	4	80.00	7	5	71.43	8.57	− 38.50–47.70	1.000
Suprasphincteric	6	2	33.33	6	2	33.33	0	− 43.64–43.64	1.000

#### Secondary endpoints

##### VAS score

**Fig. 8:**
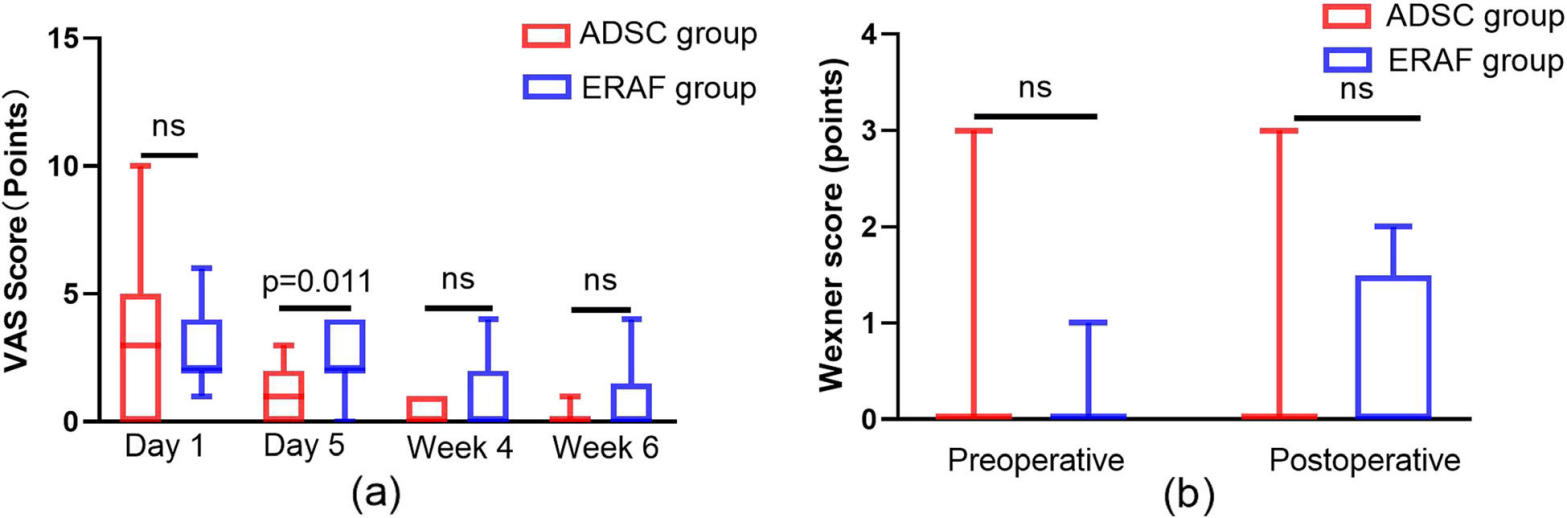
Secondary endpoints. **a** VAS score. **b** Wexner score

Although VAS score seemed lower for patients in ADSC group relative to those in ERAF group, the significant differences between the two groups was examined only at the 5th day postoperatively [3(0,5) VS 2(2,4), *p* = 0.575; 1(0,2) VS 2(2,4), *p* = 0.011; 0(0,1) VS 0(0,2), *p* = 0.332; 0(0,0) VS 0(0,1.5), *p* = 0.086; Fig. 8a].

##### Wexner score

There was no increase in Wexner score of all patients. And there were no significant differences between the groups at all follow-up points (Fig. 8b).

### Safety

Over the 12-week follow-up, there were no significant differences in the percentage of patients who experienced AEs in ADSC and ERAF group (3/11 [27.27%] and 7/13 [53.85%], respectively, difference [95% CI] 26.57 percentage points [− 11.74 to 55.45], *p* = 0.240; Table 4). None of patients withdrew from the 12-week study period due to AEs. There were no serious AEs occurred in all patients. No deaths occurred during the trail.

**Table 4:** Safety evaluation based on adverse events observed during 12-week follow-up

Contents	ADSC group (***n*** = 11)	ERAF group (***n*** = 13)
**Overall**	**3**	**7**
**AEs**		
**Perianal pain**	**3**	**1**
**Anal distention**	**0**	**7**
**Uroschesis**	**0**	**1**
**SAEs**	**0**	**0**

## Discussion

The surgical treatment of CPAF is a huge challenge for colorectal surgeons. The biggest risk is anal incontinence caused by sphincter injury. It is reported that the incidence of anal incontinence after fistulotomy of complex CPAF is up to 40% [22]. Therefore, with the continuous development of the technology, the principle of surgical treatment of fistula-in-ano has become to cure fistula-in-ano while decreasing the injury of anal function possibly [3]. As a result, emergence of various sphincter-sparing surgeries has been a trend and necessity now.

ERAF is a classic sphincter-sparing operation for the treatment of complex CPAF, and its healing rate is 66–87% [3]. So it is recommended for the treatment of fistula-in-ano by American Society of Colon and Rectal Surgeons (ASCRS) and Italian Society of Colorectal Surgery (Società Italiana di Chirurgia Colo-Rettale, SICCR) (Strong recommendation based on moderate-quality evidence, 1B) [3, 23]. Although the sphincter is not been incised, it is still reported that 13.2% of patients with CPAF suffer from mild to moderate incontinence in a systematic review, and anorectal manometry found that the anal canal resting pressure and anal canal squeeze pressures are decreased postoperatively [3, 24, 25].

The study of fistula-in-ano’s treatment with mesenchymal stem cells (MSCs) had become a research hotspot, which had a great prospect and potential future application since Garcia-Olmo et al. [26] And the American Food and Drug Administration and European Union approved CX601 (human allogeneic ADSC) for the treatment of Crohn’s fistula-in-ano in 2017 and 2018 respectively. Therefore, we believed that ADSC has a potential future application in the treatment of CPAF according to several previously published literatures (Table 1) [14–19].

Cryptoglandular infection is the key cause of CPAF, which provide a free channel for infection to pass from the anal lumen deep into the sphincter muscles [27]. Therefore, the management of internal openings to block the source of infection from the anal lumen is the key to the treatment of CPAF. It was reported that MSC can inhibit inflammation by significantly reducing the secretion of pro-inflammatory cytokines such as tumor necrosis factor (TNF) and interleukin (IL)-1β and increasing the production of anti-inflammatory cytokines such as IL-10β [28]. It was also reported that MSC can selectively migrate to the site of tissue injury and inflammation [29–31]. So, we believed that ADSC can promote tissue regeneration and repair through its paracrine, which migrates to the site of tissue injury and inflammation selectively and reduces the degree of fibrosis by inhibiting inflammatory. And it can strengthen the management of the internal opening to block the source of infection from the anal lumen.

To our knowledge, this is the first report of a study in which ADSCs were used to treat CPAF in China. Our study indicated that the closure rate of ADSC was not inferior to that of ERAF at week 12 (54.55% VS 53.85%, *p* = 1.000). And it was similar to some other studies [15, 19]. As Cho et al. [32] and Choi et al. [17] reported, we also thought that some failure of the cases with ADSC in this study may be caused by poor control of infection. However, we supposed that the principal reason is the lack of the experience of operation, because we did not have any reference of procedures due to the first study in China. In addition, it seems to be lower that the short-term closure rate compared with our previous study in the patients with Crohn’s disease [20], which may be caused by the small sample size.

Minimally invasive surgery is a trend of fistula-in-ano’s surgical treatment, which focuses on enhanced recovery and functional protection. On the one hand, the results showed that the postoperative pain of patients was significantly decreased on the 5th day and almost returns to normal at week 4. Although there was no significant difference in VAS score between the two groups due to the limitation of sample size, and VAS score of ADSC group was higher than that of ERAF group at day 1 because a patient reported his score was 10. We still believed that ADSC has an advantage in pain relief. On the other hand, there was no incontinence due to the injury of sphincter in the study as previously literatures reported (Table 1) [14–19]. The damage of anal function after the operation of ERAF may be related to the stretch injuries to the muscle and nerve fibers caused by significant retraction and manipulation of the anus [33]. However, ADSC can protect the anal sphincter better because it does not need significant retraction and manipulation of the anus compared with ERAF.

Autologous ADSC can avoid autoimmune rejection and ensure the safety of treatment in this study. The study showed that ADSC is well tolerated and there was no evidence of specific adverse events associated with it, which was consistent with the results of other studies [14–19]. The results showed that more than half of the patients in ERAF group appear anal distention in the early postoperative period, which may be related to the retraction, manipulation, and suture of flap, while there was no anal distention in ADSC group and the patients felt more comfortable after operation.

The main limitation in this study had been that small sample size and no randomization have an impact on the results. On the one hand, only 24 cases were enrolled in our study until the end of 2019 because it could not go on this year for various reasons. On the other hand, we had to ask the patients’ desire before liposuction because autologous ADSC was used in the study and could not randomize due to medical inform, patients’ informed consent, and relevant laws in China. We will conduct large scale randomized controlled trials (RCTs) to evaluate the safety and efficacy of ADSC in the future. Another limitation in this study would be that the follow-up time is short, and only 12 weeks follow-up results were reported in this article due to the short follow-up of some cases. We will continue our follow-up and report the results in the future. Otherwise, high cost of ADSC preparation and two operations limit the promotion and application of ADSC in some degree as we have reported before [20].

In summary, we observed that ADSC is a safe and effective treatment which is not worse than ERAF in the treatment of complex CPAF. It can protect anal function of patients, relieve pain, reduce complications, recover quickly, and provide good tolerance.

## Conclusion

This study indicated safety and efficacy of ADSC for the treatment of complex CPAF in the short term, which is not inferior to that of ERAF. ADSC may provide a promised and potential treatment for complex CPAF conforming to the future of the treatment, which is reconstruction and regeneration.

## Data Availability

The datasets used and/or analyzed during the current study are available from the corresponding author upon request. After the completion of the trial, all data will be uploaded to China clinical trial registration center.

## Abbreviations

ERAF: Endorectal advancement flap
ADSC: Adipose-derived stem cells
CPAF: Cryptoglandular perianal fistula
MRI: Magnetic resonance imaging
ERUS: Endorectal ultrasonography
VAS: Visual analog score
AE: Adverse event
SAE: Serious adverse event
ASCRS: American Society of Cataract and Refractive Surgery
SICCR: Società Italiana di Chirurgia Colo-Rettale
MSC: Mesenchymal stem cells
TNF: Tumor necrosis factor
IL: Interleukin
RCT: Randomized controlled trials
